# Predicting the effect of sulfadoxine-pyrimethamine uptake by pregnant women on birth weight using a generalized ordered partial proportional odds model

**DOI:** 10.1186/s12884-022-04565-7

**Published:** 2022-03-19

**Authors:** Chris Guure, Seth Afagbedzi

**Affiliations:** Department of Biostatistics, School of Public Health, University of Ghana, Legon-Accra, Ghana

**Keywords:** Sulphadoxine-pyrimethamine, Low birth weight, Normal birth weight, Macrosomia, Pregnant women, Predictive probability, Generalized ordered partial proportional odds model

## Abstract

**Background:**

Low birth weight is a public health problem in Africa with the cause attributable to malaria in pregnancy. World Health Organization recommends the use of intermittent preventive treatment in pregnancy (IPTp) with sulfadoxine-pyrimethamine to prevent malaria during pregnancy. The objective of this study was to evaluate the prevalence and trajectories of birth weight and the direct impact and relationship between sulfadoxine-pyrimethamine and birth weight in Ghana since 2003.

**Method:**

This study used secondary data obtained from the Demographic and Health Survey conducted in Ghana since 2003. Low birth weight was defined as weight < 2500 g irrespective of the gestational age of the foetus, while normal birth weight was between 2500 g to < 4000 g and macrosomia was =  > 4000 g. In all the analysis, we adjusted for clustering, stratification and weighting to reduce bias and improve precision of the estimates. Analysis was performed on each survey year as well as the pooled dataset. The generalized ordered partial proportional odds model was used due to violations of the parallel regression model assumptions. Efforts were made to identify all confounding variables and these were adjusted for. Predictive analysis was also executed.

**Results:**

The overall prevalence of low birth weight was 9% while that of macrosomia was 13%. The low birth weight for 2003 was 12% while in 2008 it was 21% and then 68% in 2014. The mean birth weight of the children in 2014 was 3.16 (3.14, 3.19), 2008 was 3.37 (3.28, 3.45) and 2003 was 3.59 (3.49, 3.69) while that of the pooled data was 3.28 (3.25, 3.30). The adjusted model (taking into consideration all confounding variables) showed that non-uptake of SP could result in 51% odds of giving birth to a low-birth-weight compared with normal birth weight child. An insignificant result was observed between macrosomia and low birth weight.

**Conclusion:**

There is higher probability that low birth weight could increase over the next couple of years if measures are not taking to reverse the current trajectories. The uptake of sulfadoxine-pyrimethamine should continue to be encouraged and recommended because it has a direct beneficial effect on the weight of the child.

## Background

Malaria remains a major public health threat to people in the African continent. In 2018, 213 out of 228 million cases of malaria recorded worldwide, occurred in the African Region, World Health Organization (WHO) [1]. The Region accounted for 94% of 405,000 global malaria deaths in 2018. The at most risk groups of malaria infection include pregnant women, infants, and children under-five years of age [2]. About 11 million pregnancies in moderate and high transmission sub-Saharan African countries would have been exposed to malaria infection in 2018, with prevalence of exposure to malaria infection in pregnancy being as high as 35%. Maternal, infant and child health consequences of malaria have long been recognized [3–12]. 

The harmful effects of malaria in pregnancy on the morbidity and mortality of foetuses and newborns have been of grave concern to many. Studies have shown maternal anaemia [3, 4] and placental insufficiency [5–7] as the main effects of malaria in pregnancy, which together result in either preterm delivery [8, 9] or intrauterine growth restriction [10]. The outcome of which is low birth weight of neonates born to malaria infected mothers [11, 12]. There are more than 20 million babies born annually with low birth weight majorly caused by malaria in pregnancy [13]. Approximately, 96% of newborns are in the developing nations with about 22% based in Africa [13].

Birth weight can be classified into three: Low birth weight, normal birth weight and high birth weight (macrosomia). Low birth weight has been defined as weight less than 2500 g irrespective of the gestational age of the foetus while normal weight is between 2500 g to less than 4000 g and macrosomia is weight greater than or equal to 4000 g [13].

Birth weight is a serious public health problem in sub-Saharan Africa based on which a number of interventions have been rolled out to mitigate if not eradicate it completely and one of such interventions is the uptake of sulfadoxine-pyrimethamine in pregnancy. Intermittent preventive treatment of malaria in pregnancy (IPTp) with sulfadoxine-pyrimethamine is an antimalaria medication which is among a package of interventions recommended by the WHO for controlling malaria and its effects during pregnancy irrespective of whether the pregnant woman is infected or not. The recommended IPTp drug is sulfadoxine-pyrimethamine (WHO) [14]. According to a WHO evidence review [15], three or more doses of IPTp-SP were associated with higher mean birth weight and fewer low birth weight (LBW) for both the mother and the unborn child. Similarly, a study conducted by Kayentao et al. [16], showed a strong relationship between two or more doses of IPTp-SP and malaria related outcomes as well as sexually transmitted infections/ reproductive tract infections. It has been recommended by WHO that the first dose of IPTp-SP in malaria endemic areas be administered in the early stages of the second trimester (13 to 16 weeks) of a pregnancy, World Health Organization [17]. Further recommendation is made for pregnant women to receive IPTp-SP during subsequent visits to a health facility by a health care provider. The expected benefit of the use of IPTp-SP is prevention of the adverse consequences of malaria on maternal and foetal outcomes, such as placental infection, clinical malaria, maternal anaemia, fetal anaemia, low birth weight and neonatal mortality [9].

There have been a number of studies conducted to look at the causes and variables associated with birth weight, some of which include preterm birth and intrauterine growth restriction [18–21]. A study conducted by Isiugo-Abanihe and Oke [22], found that pre-term birth, maternal age and maternal malnutrition are associated with low birth weight. Lu et al., [23] showed that macrosomia prevalence rate is increasing, from 2–20% in the developing countries, 6.0–7.8% in China and about 10.9% in Ghana [24]. It is expected that as the prevalence of overweight and obesity increases in the developing countries, there will be a corresponding increase in the prevalence of macrosomia [25, 26].

Ghana started full implementation of IPTp policy in 2005 after its adoption in 2003. After more than a decade of IPTp policy implementation, evidence-based analysis of the accumulated data is critical to inform the need to scale-up the programme. This has informed the current study with the objective to predict the direct effect of sulfadoxine-pyrimethamine uptake among pregnant women on birth weight in Ghana using the Demographic and Health Survey data.

## Methodology

### Source of data

This study is conducted using data obtained from the Demographic and Health Surveys (DHS) program that implements population and health surveys in several countries across the world. These are nationally representative household surveys which provides information on a number of indicators in maternal and child mortality, reproductive health, HIV, family planning and others. A data request was submitted to the official DHS website. The request was approved and dataset was provided via email within two working days. Data for the standard DHS was obtained and on three survey years (2003, 2008 and 2014).

### Sample design and population

Demographic and Health Surveys employ a stratified two-stage sampling design. The first stage involves the selection of clusters also referred to as primary sampling units which in some cases may be the same or a number constituting an enumeration area usually drawn from the most recent census data. These clusters are systematically selected based on probability proportionate to size. At the second stage, a sampling frame made up of listing of all households within a cluster is obtained. This is to allow for equal chances of selection and to make the data obtained nationally representative. Households to be included in the survey are then selected systematically from the sampling frame. In the selected households, only women between the ages of 15–49 years who were either permanent residents or stayed over the night before the survey were eligible to be interviewed. The variables for this analysis were extracted from the 2003, 2008, and 2014 Ghana Demographic and Health Survey’s (GDHS) structured questionnaires.

### Data collection tool

The DHS used a structured questionnaire as an interview guide for data collection. Different types of questionnaires classifications were used to collect the data namely household, women, men, children and biomarker. This study utilised the women’s survey in which data was collected from all eligible women between the ages of 15 and 49 concerning topics such as socio-demographic characteristics, women characteristics, birth history, IPTp-SP uptake, birth weight amongst others.

### Data variables

#### Dependent variable

The dependent variable for this study is birth weight (at birth) which is defined and categorised as follows: low birth weight (birth weight < 2.5 kg), normal (birth weight ≥ 2.5 kg < 4.0 kg) and high (macrosomia—birth weight ≥ 4.0 kg) birth weight. It was coded as an ordinal outcome. A code of “1” was assigned to children who were classified as low birth weight, “2” as normal birth weight and “3” macrosomia.

#### Primary exposure variables

The primary exposure of interest was the uptake of sulfadoxine-pyrimethamine (fansidar). The World Health Organization recommends that sulfadoxine-pyrimethamine (SP) be given to all pregnant women starting as early as possible in the second trimester (i.e., not during the first trimester). In this study, the uptake of SP was defined as pregnant women who took at least one dose of sulfadoxine-pyrimethamine. Pregnant women who received at least one dose of SP or fansidar were categorized as “yes” and assigned “1” and those who did not at all were classified as “no” and assigned “2”.

#### Confounding variables

All other independent variables (confounders) were grouped into four. These were maternal factors (body mass index, number of ANC visits, months of pregnancy at first ANC, gravidity, told about pregnancy complications, age at birth, occupation, educational status). Child factors (birth order, sex of the child, preceding birth interval). The third category was socio-economic (place of residence, wealth index, marital status). The fourth was the combination of maternal factors, child factors and socio-economic factors.

### Statistical analysis

The generalized ordered partial proportional odds model is used to estimate the odds ratios of the predictor on the predicted of interest controlling for identified confounding variables for each survey year and that of the pooled dataset. The model can be specified as follows:

1 $$P\left({Y}_{i}>j\right)=g\left(X{\beta }_{j}\right)= \frac{\mathrm{exp}({\alpha }_{j}+{X}_{i}{\beta }_{j})}{1+\left\{\mathrm{exp}\left({\alpha }_{j}+{X}_{i}{\beta }_{j}\right)\right\}}, j=1, 2, . . . , M-1$$

where *M* represents the number of categories of the ordinal variable, in this case three (3), that is, low birth weight, normal and macrosomia birth weights. It can therefore be deduced from Eq. (1) that the probability that $Y$ will take on each value say, $1, . . . , M$ are equal to

2 $$P\left({Y}_{i}=1\right)=1-g({X}_{i}{\beta }_{1})$$

3 $$P\left({Y}_{i}=j\right)=g\left({X}_{i}{\beta }_{j-1}\right)- g\left({X}_{i}{\beta }_{j}\right) j=2, . . . , M-1$$

4 $$P\left({Y}_{i}=M\right)=g({X}_{i}{\beta }_{M-1})$$

Based on these expressions, if *M* = *2*, the generalized ordered partial proportional odds model is equivalent to the standard logistic regression model.

Equation (1) is similar to a parallel-lines model except that in the parallel-lines (proportional odds) model all *β*’*s* are constrained to be the same for all values of *j* thereby being equivalent to

5 $$P\left({Y}_{i}>j\right)=g\left(X\beta \right)= \frac{\mathrm{exp}({\alpha }_{j}+{X}_{i}\beta )}{1+\left\{\mathrm{exp}\left({\alpha }_{j}+{X}_{i}\beta \right)\right\}}, j=1, 2, . . . , M-1$$

The key issue with the parallel-lines model which is mostly used is as a result of its restrictive assumption on the *β*’*s* which are likely to be violated due to differences across the values of *j’s*. These restrictive assumptions are easily overcome with the use of the proposed generalized ordered partial proportional odds model [27]. This model was used to find the statistical association between birth weight (low birth weight, normal birth weight and macrosomia) and the primary predictor (SP uptake) of interest controlling for identified confounding variables for each survey year and that of the pooled dataset.

In all the analysis, we adjusted for the complex nature of the survey design by accounting for clustering, stratification and weighting to reduce bias and improve the precision of the estimates. Analysis was performed on each survey year as well as the pooled dataset. Efforts were made to identify all confounding variables and these were adjusted for. Variables that were included in the adjusted model were those that showed significance at the bivariate level was maintained only if it influenced the estimate of the primary exposure variables or the outcome or both (using a 20% threshold of type I error). A type I error of 5% was specified and used for variables significance and interpretation.

All analyses were based on complete cases, where any observation with missing variable(s) was dropped. This was adopted because the samples were large enough to show any differences between groups if it indeed exists. All analyses were carried out with Stata version 17.

### Ethics approval and consent to participate

The DHS surveys have been reviewed and approved by International Classification of Functioning, Disability and Health (ICF) and Ghana Health Service Institutional Review Boards (IRB). The ICF IRB approved the study protocol, survey instruments and materials prior to the study commencement. This ensures that the survey complies with the U.S. Department of Health and Human Services regulations for the protection of human subjects. Verbal informed consent was sought from DHS respondents to all the questionnaires as well as drawing of blood in surveys. Further to that was a verbal informed consent sought by the interviewer reading a prescribed statement to the respondent and recording in the questionnaire whether the respondent consented (or provided assent on behalf of minors). Interviewer then signed his or her name attesting to the fact that he/she read the consent statement to the respondent and they agreed to participate.

## Results

### Descriptive

**Fig. 1:**
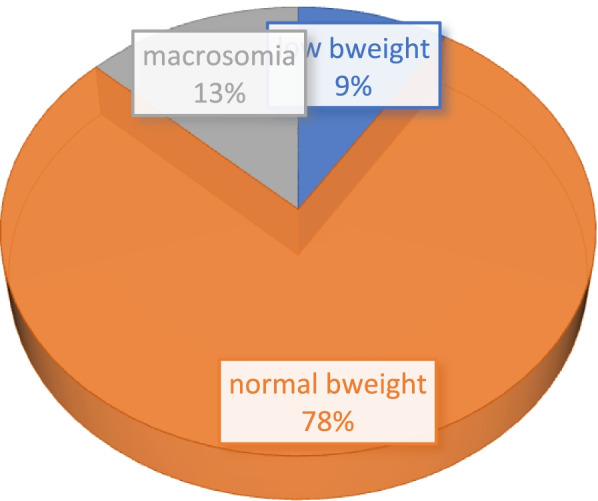
percentage distribution of birth weight over the three survey periods in Ghana

**Fig. 2:**
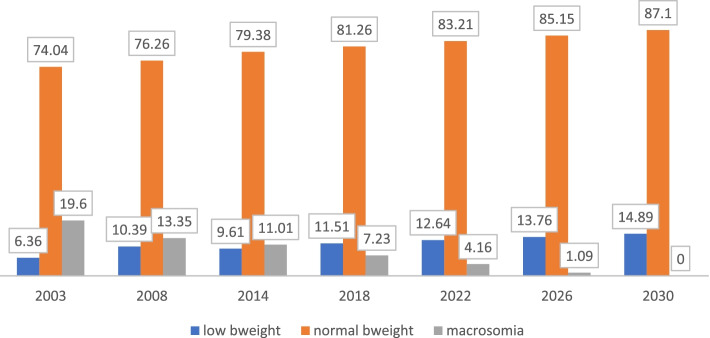
Forecasting birth weight (%) in Ghana based on the 2003, 2008 and 2014 DHS surveys

**Fig. 3:**
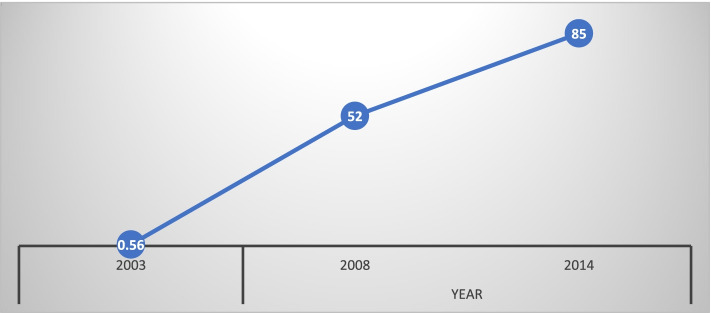
Illustration of Fansidar/SP uptake during pregnancy over the three survey periods

The overall prevalence of low birth weight is 9% while that of macrosomia is 13% Fig. 1. The low birth weight for 2003 was 6.36% while in 2008 it was 10.39% and then 9.61% in 2014, Fig. 2. For macrosomia, Ghana recorded 19.60% in 2003, 13.35% in 2008 and 11.01% in 2014. It is therefore projected that by the year 2030, macrosomia will decrease to about 0% while that of low birth weight will increase to about 14.89 percentage points from its current rate if measures are not taken to mitigate the current trajectories, Fig. 2. There is an upward trajectory of the uptake of SP since 2003, that is from 0.56% to as high as 85%, Fig. 3. The mean birth weight of the children in 2014 was 3.16 kg (3.14 kg, 3.19 kg), 2008 was 3.37 kg (3.28 kg, 3.45 kg) and 2003 was 3.59 kg (3.49 kg, 3.69 kg) while that of the overall analysis was 3.28 kg (3.25 kg, 3.30 kg).

### Inferential

We used the brant test as provided by Long and Freese’s “spost” routines to first of all determine whether there was any violation of the parallel regression assumption that made it impossible to apply the standard ordered proportional odds model [27]. The test result gave a global *p*-value < 0.001 with a corresponding test statistic of 42.52. This indicates highly significant evidence that the parallel regression assumption has been violated. The main primary exposure variable (SP uptake) failed the parallel regression assumption. Other variables included child sex, preceding birth interval and mother’s BMI. As a result of the violation, we used the generalized ordered partial proportional odds model.

The results as presented in Table 1, shows that the crude odds ratio for pregnant women who do not take SP is 45 (OR:1.45) percentage points higher in giving birth to low birth weight as against normal birth weight. This is statistically significant with a confidence interval of 1.19 to 1.78. The adjusted model (taking into consideration all confounding variables at a statistical significance level of 5%) shows that non-uptake of SP could result in 51% odds of giving birth to a low-birth-weight child. Also, from the same analysis, it is observed that non-uptake of SP could result in 15% odds of given birth to a macrosomia child compared to low birth weight with the unadjusted (crude) generalized ordered proportional odds model. That of the adjusted model shows that non-uptake of an SP results in giving birth to a low-birth-weight child compared to a macrosomia with an OR of 0.94 and a confidence interval of 0.54 to 1.63, which is statistically insignificant, Table 1. Overall, there were only three significant confounding variables (child sex, number of ANC visits and preceding birth interval) in the final adjusted model.

**Table 1:** crude and adjusted odds ratios of sulfadoxine-pyrimethamine on birth weight using the pooled dataset

Variables	Unadjusted Model	Child Characteristics	Mother Characteristics	Socio cultural and Economic	Final Model
**NORMAL VS LOW BIRTH WEGHT**					
Fansidar sp (non-uptake)	1.45(1.19,1.78)*	1.48(1.11,1.96)*	1.41(1.09,1.81)*	1.47(1.20,1.80)*	1.51(1.08,2.13)*
Child sex		0.92(0.73,1.18)			0.93(0.67,1.26))
Birth order no		1.02(0.94,1.11)			1.03(0.72,1.48)
Pre birth interval		1.00(1.00,1.00)			1.00(0.99,1.00)
Number of anc visit			0.71(0.55,0.91)*		0.59(0.44,0.79)*
Months of preg. At first anc			0.98(0.91,1.06)		0.94(0.86,1.03)
No. of children ever born			1.06(0.97,1.15)		1.00(0.74,1.36)
Told about preg. Complications			0.94(0.70,1.26)		1.09(0.74,1.62)
Age at birth			1.00(0.97,1.03)		1.00(0.96,1.03)
Resp. bmi			1.01(0.98,1.04)		1.01(0.98,1.04)
Maternal Educ			1.05(0.90,1.23)		1.11(0.92,1.35)
Resp. occupation			0.99(0.94,1.04)		1.02(0.96,1.09)
Place of resident				0.82(0.65,1.02)	0.75(0.53,1.07)
Wealth index				0.96(0.89,1.03)	1.03(0.91,1.16)
Marital status				0.93(0.76,1.08)	1.14(0.77,1.68)
**MACROSOMIA VS LOW BIRTH WEGHT**					
Fansidar sp (non-uptake)	0.85(0.62,1.16)	1.24(0.81,1.91)	0.72(0.49,1.05)	0.88(0.65,1.21)	0.94(0.54,1.63)
Child sex		0.49(0.34,0.71)*			0.58(0.36,0.92)*
Birth order no		1.05(0.95,1.18)			1.17(0.62,2.18)
Pre birth interval		1.00(1.00,1.01)			1.01(1.00,1.01)
Number of anc visit			0.71(0.51,0.98)		0.51(0.32,0.82)*
Months of preg. at first anc			0.96(0.87,1.06)		0.92(0.80,1.06)
No. of children ever born			0.96(0.83,1.11)		0.96(0.57,1.61)
Told about preg. complications			1.01(0.67,1.55)		1.03(0.55,1.93)
Age at birth			1.02(0.97,1.06)		1.00(0.93,1.07)
Resp. bmi			0.98(0.93,1.03)		0.95(0.90,1.01)
Maternal Educ			1.17(0.91,1.49)		1.13(0.85,1.49)
Resp. occupation			1.01(0.94,1.09)		1.05(0.95,1.16)
Place of resident				1.05(0.77,1.43)	1.29(0.70,2.36)
Wealth index				0.98(0.88,1.09)	1.12(0.91,1.37)
Marital status				0.88(0.64,1.21)	1.37(0.78,2.42)

Asterisks (*) indicates a p-value < 0.05 significance level

Presented in Tables 2 and 3 are results according to 2008 and 2014 survey years respectively. Though in 2008, there is 15% increase odds of given birth to low birth weight as compared to normal birth weight while 45% have a higher odd of being born macrosomia as against low birth weight for non-uptake of SP, none was statistically significant. The 2014 survey year showed a much higher odds (OR: 1.38, 95% CI – 0.78, 2.44) and (OR: 1.96, 95% CI- 0.81, 4.73) of being born with a low birth weight compared to normal and macrosomia birth weights for non-uptake of SP respectively.

**Table 2:** crude and adjusted odds ratios of sulfadoxine-pyrimethamine on birth weight using the 2008 dataset

Variables	Unadjusted Model	Child Characteristics	Mother Characteristics	Socio cultural and Economic	Final Model
**NORMAL VS LOW BIRTH WEGHT**					
Fansidar sp (non-uptake)	1.07(0.74,1.61)	1.07(0.68,1.84)	1.10(0.72,1.69)	0.95(0.63,1.42)	1.15(0.70,1.88)
Child sex		1.16(0.73,1.84)			1.12(0.69,1.81))
Birth order no		1.01(0.86,1.17)			0.91(0.51,1.62)
Pre-birth interval		1.00(0.99,1.01)			1.00(0.99,1.01)
Number anc visit			0.58(0.37,0.88)*		0.59(0.39,0.90)*
Months of preg. at first anc			0.95(0.84,1.08)		0.91(0.80,1.04)
No. of children ever born			1.08(0.92,1.26)		1.10(0.70,1.74)
Told about preg. complications			0.77(0.49,1.23)		0.93(0.57,1.50)
Age at birth			0.99(0.95,1.04)		0.98(0.92,1.03)
Resp. bmi			1.01(0.97,1.05)		1.01(0.97,1.05)
Maternal Educ			1.09(0.83,1.44)		1.09(0.81,1.47)
Resp. occupation			1.03(0.92,1.14)		1.06(0.95,1.18)
Place of resident				0.91(0.56,1.47)	0.94(0.56,1.58)
Wealth index				0.90(0.77,1.05)	0.86(0.72,1.02)
Marital status				1.27(0.87,1.85)	1.17(0.63,2.17)
**MACROSOMIA VS LOW BIRTH WEGHT**					
Fansidar sp (non-uptake)	0.54(0.28,1.03)	0.68(0.34,1.37)	0.54(0.27,1.07)	1.06(0.73,1.59)	0.55(0.24,1.28)
Child sex		0.66(0.29,1.52)			0.64(0.27,1.53)
Birth order no		1.03(0.81,1.30)			1.16(0.26,5.20)
Pre birth interval		1.01(1.00,1.01)			1.01(0.99,1.02)
Number anc visit			0.62(0.36,1.07)		0.45(0.21,0.97)
Months of preg. at first anc			0.77(0.63,0.95)*		0.70(0.56,0.89)*
No. of children ever born			1.03(0.81,1.30)		0.95(0.29,3.13)
Told about preg. complications			0.50(0.26,0.96)*		0.58(0.19,1.78)
Age at birth			1.04(0.97,1.11)		0.99(0.89,1.11)
Resp. bmi			1.00(0.93,1.07)		0.97(0.88,1.06)
Maternal Educ			0.95(0.62,1.46)		0.90(0.51,1.59)
Resp. occupation			1.09(0.95,1.24)		1.13(0.97,1.31)
Place of resident				1.30(0.63,2.71)	2.02(0.72,5.71)
Wealth index				0.90(0.69,1.17)	0.97(0.75,1.27)
Marital status				1.23(0.75,2.01)	1.24(0.42,3.70)

Asterisks (*) indicates a p-value < 0.05 significance level

**Table 3:** crude and adjusted odds ratios of sulfadoxine-pyrimethamine on birth weight using the 2014 dataset

Variables	Unadjusted Model	Child Characteristics	Mother Characteristics	Socio cultural and Economic	Final Model
**NORMAL VS LOW BIRTH WEGHT**					
Fansidar sp (non-uptake)	1.40(0.97,2.03)	1.38(0.88,2.17)	1.47(0.86,2.50)	1.41(0.97,2.04)	1.38(0.78,2.44)
Child sex		0.87(0.65,1.15)			0.89(0.60,1.31))
Birth order no		1.03(0.94,1.14)			1.23(0.80,1.88)
Pre birth interval		1.00(1.00,1.00)			1.00(0.99,1.00)
Number anc visit			0.73(0.50,1.07)		0.62(0.42,0.92)*
Months of preg. at first anc			0.97(0.87,1.07)		0.96(0.85,1.08)
No. of children ever born			1.07(0.93,1.22)		0.89(0.60,1.30)
Told about preg. complications			1.18(0.71,1.96)		1.55(0.83,2.89)
Age at birth			1.00(0.96,1.03)		1.01(0.96,1.05)
Resp. bmi			1.02(0.98,1.06)		1.03(0.98,1.08)
Maternal Educ			1.18(0.96,1.46)		1.09(0.85,1.40)
Resp. occupation			0.98(0.91,1.06)		1.00(0.92,1.08)
Place of resident				0.92(0.70,1.21)	0.55(0.34,0.90)*
Wealth index				1.01(0.92,1.10)	1.18(0.99,1.40)
Marital status				0.96(0.73,1.28)	0.89 (0.54,1.48)
**MACROSOMIA VS LOW BIRTH WEGHT**					
Fansidar sp (non-uptake)	1.54(0.91,2.60)	1.60(0.82, 3.12)	1.49(0.79,2.79)	1.52(0.89,2.58)	1.96(0.81,4.73)
Child sex		0.47(0.31,0.71)			0.50(0.25,1.02)
Birth order no		1.04(0.91,1.19)			0.96(0.53,1.73)
Pre birth interval		1.00(1.00,1.01)			1.00(1.00,1.01)
Number anc visit			0.73(0.45,1.17)		0.47(0.23,0.94)*
Months of preg. at first anc			1.00(0.89,1.12)		1.10(0.89,1.36)
No. of children ever born			0.90(0.73,1.12)		1.02(0.60,1.74)
Told about preg. complications			1.32(0.70,2.50)		1.97(0.72,5.36)
Age at birth			1.02(0.97,1.08)		1.02(0.91,1.14)
Resp. bmi			0.96(0.92,1.00)		0.93(0.85,1.02)
Maternal Educ			1.34(0.95,1.90)		1.13(0.77,1.68)
Resp. occupation			1.00(0.91,1.10)		1.05(0.92,1.20)
Place of resident				1.03(0.72,1.47)	1.16(0.52,2.61)
Wealth index				1.02(0.91,1.14)	1.21(0.89,1.65)
Marital status				0.85(0.56,1.30)	1.87(0.70,4.99)

Asterisks (*) indicates a p-value < 0.05 significance level

**Fig. 4:**
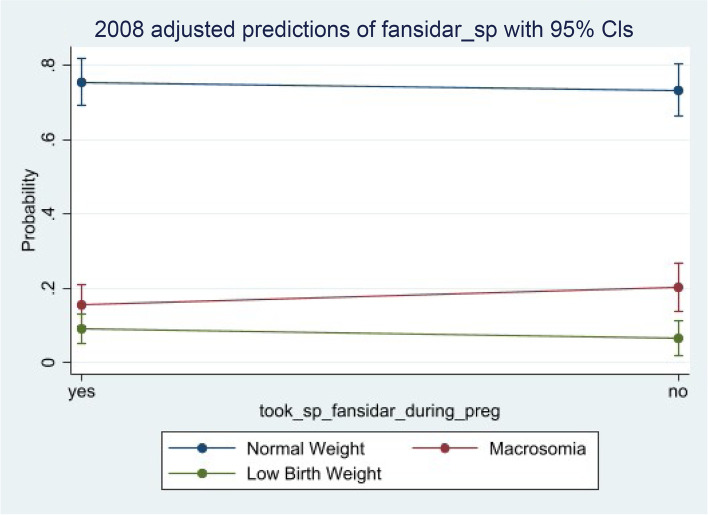
Predictive probabilities of sulfadoxine-pyrimethamine holding all the confounders at their means for 2008

**Fig. 5:**
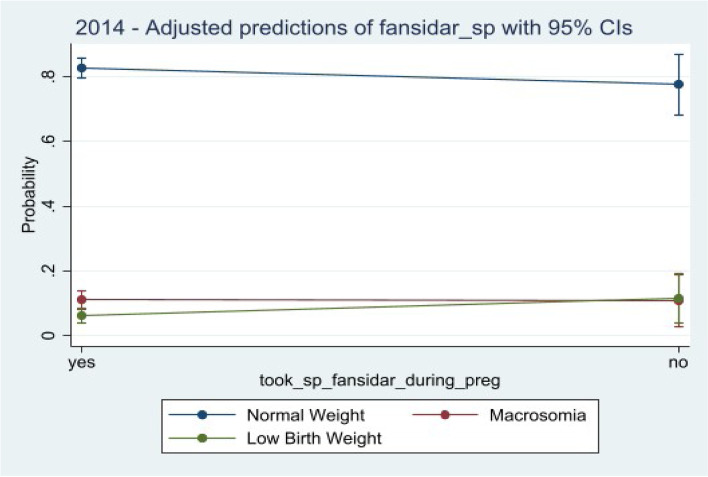
Predictive probabilities of sulfadoxine-pyrimethamine holding all the confounders at their means for 2014

**Fig. 6:**
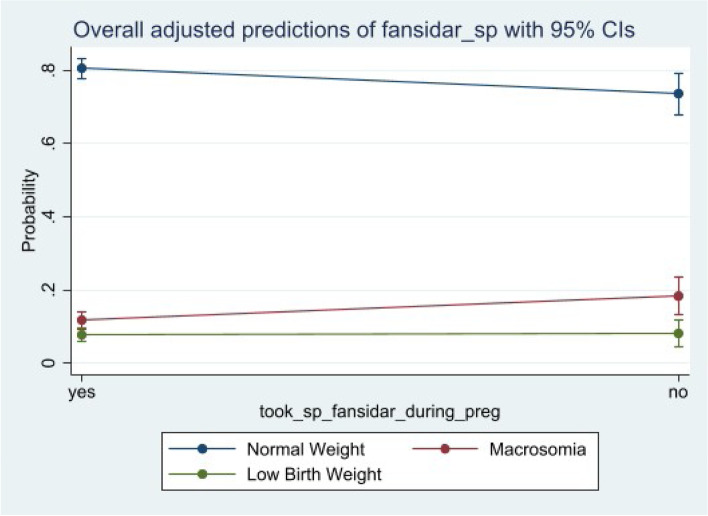
Predictive probabilities of sulfadoxine-pyrimethamine holding all the confounders at their means for overall

Figure 4 presents the adjusted predictive model of SP uptake with their corresponding confidence intervals for the overall data. An observation of the figure shows that pregnant women who take SP have a higher probability of giving birth to normal weight children while those who do not or said they did not, have a decrease or lower probability of giving birth to a normal weight child. Pregnant women who had not taken SP at the time of pregnancy had an increase probability of giving birth to low-birth-weight children in 2014 (Fig. 5) and macrosomia children in 2008 (Fig. 4) and the overall (Fig. 6), though it was much higher among macrosomia than among low birth weight, Fig. 6.

## Discussion

This is the first study of its kind to have looked at the three main birth weights in Ghana using a nationally representative survey (DHS) from 2003 to 2014 with an approximately five years interval. The study examines the prevalence of birth weight (low, normal and macrosomia birth weights) and their projected trajectories by the end of 2030. The study goes further to establish a relationship between birth weight and SP uptake during pregnancy by controlling for all relevant and available confounders. In this study, we established the need to apply a generalized ordered partial proportional odds model due to violations of the parallel regression model assumptions using the brant test.

The findings show that over the three survey years, the prevalence of macrosomia is higher than that of low birth weight. Specifically, there was a higher prevalence of macrosomia in all the survey years (2003, 2008 and 2014) than there was low birth weight. We observed a higher projection of low birth weight taking into consideration the previous year’s prevalence to 14.89 percentage increase while that of macrosomia will decrease to about zero (0) percentage points. The higher projection of low birth weight maybe as a result of the increase in prevalence observed in the 2008 and 2014 over 2003 survey years. The higher prevalence of low birth weight may be attributed to poor socioeconomic status, maternal malnutrition and inadequate antenatal care as reported elsewhere [28–31]. This should be of much concern to policy makers and implementers. The overall prevalence of low birth weight is in sync with a finding reported by [32].

The high prevalence of macrosomia as observed in this study confirm findings of other studies in some developing countries such as Nigeria, Uganda and Algeria that report a prevalence of 7.5, 8.4 and 14.9 percentage points respectively [33]. Another study in China, revealed an increasing prevalence of macrosomia from 6.0% to 7.8% over a 10-year period [23]. A similar high prevalence of macrosomia (10.9%)in support of this findings have been reported elsewhere [24]. Ro, Goldberg and Kane, 2019 [34] reports higher prevalence of macrosomia among non-white Hispanic (11.41%) and Hispanic (5.29%) as against low birth weight for non-white Hispanic (3.92%) and Hispanic (5.29%) respectively.

This study further reveals the importance of pregnant women taking sulfadoxine-pyrimethamine during pregnancy to help improve the weight of their new born. It shows that pregnant women who took SP during pregnancy were more likely to give birth to normal weight children than to low-birth-weight children when the two were compared. We moved further to look at predictive probabilities of pregnant women who did not take sulfadoxine-pyrimethamine and realized that taking SP decreases the probability by about 7.4 (1.1 – 13.8) percentage points of giving birth to normal weight children. The predictive probability of giving birth to a low-birth-weight child for those who did not take SP increased by about 7.4 (1.07 – 13.8) percentage points while that for macrosomia increases at a lower point of 0.06%. Both were evaluated with the full model (where confounders were controlled for) at their means and statistically significant. These findings confirm what was observed elsewhere, where increased birth weight was significantly associated with an increase SP uptake [35]. Quakyi et al. [35], found out that an uptake of three or more doses of SP results in an increased body weight by 0.165 kg. Other studies have also observed that plasmodium falciparum infection during pregnancy is a major cause of low birth weight with sulfadoxine-pyrimethamine being beneficial [16, 36].

## Conclusion

There is higher probability that low birth weight could increase over the next couple of years if measures are not taking to reverse the current trajectories. The uptake of sulfadoxine-pyrimethamine should continue to be encouraged and recommended because it has a direct beneficial effect on the weight of the child. Overall, the average body weights of children in Ghana are majorly within the WHO recommendation. Interpretation and conclusion of the findings should be made with caution due to our inability to adjust for other important variables that have the potential to influence the final results.

## Strengths and limitations

One of the major strengths of this study is the use of a nationally representative sample across Ghana over a 15-year period. The second one is the use of a more robust and appropriate statistical model called generalized ordered partial proportional odds model when the parallel regression model assumption is violated. There are a number of limitations, the first is our inability to model the primary exposure variable with the outcome of interest for the year 2003 due to insufficient sample size. In addition, the sample size used for the overall analysis was inadequate due to the proportion of women who respondent to whether they have used SP during their last pregnancy. Therefore, there were a number of missing observations and so this has the potential of affecting the conclusions. Due to the 5-year retrospective nature of the survey, the probability of recall bias affecting the data collected and subsequently the analysis and interpretation is very high. Important variables such as Neonates born with congenital defect or genetic conditions and also gestational diabetes which are more likely to affect birth weight were not obtained and therefore not adjusted for.

## Data Availability

An application requesting for the use of the Demographic and Health Surveys data was sent to the DHS website. Data was then used after approval was obtained. The datasets generated and/or analyzed during the current study are publicly available in the Demographic and Health Survey Repository, http://dhsprogram.com/data/available-datasets.cfm
